# Bushy‐Crested Hornbills Successfully Hunting Flying Bats in Gomantong Caves, Malaysia

**DOI:** 10.1002/ece3.71744

**Published:** 2025-07-04

**Authors:** José L. Tella, Cristina Fuentes‐Sendín, Carlos Gutiérrez‐Expósito, Gema Ruiz‐Jiménez, Raquel Sainz‐Elipe, Cristina B. Sánchez‐Prieto, David Serrano

**Affiliations:** ^1^ Department of Conservation Biology and Global Change Doñana Biological Station (EBD‐CSIC) Sevilla Spain; ^2^ Independant Biologist Lora del Río Spain; ^3^ RIFCON GmbH Hirschberg Germany; ^4^ Independant Biologist Bollullos de la Mitación Spain; ^5^ Department of Zoology University of Granada Granada Spain

**Keywords:** bat nocturnal behavior, bat predators, bat roosts, hornbill diet

## Abstract

Hornbills living in tropical forests are predominantly frugivorous, but some species incorporate small animals into their diets, and bats have only been anecdotally recorded among their prey. However, it is not well known how they are captured and how often. We observed bushy‐crested hornbills ( *Anorrhinus galeritus* ) capturing wrinkle‐lipped free‐tailed bats (*Mops plicatus*) in flight as thousands of them emerged from a large cave‐roost in Bornean Malaysia. At least eight individuals successfully hunted flying bats by perching on dry branches hanging from the main entrance of the cave, using two tactics: (1) by jumping and making short flights until catching the flying bats (i.e., hawking), and (2) perching, waiting for bats that fly by at short distances, catching them with quick movements of the beak (i.e., snatching). This does not appear to be an anecdotal behavior, but rather one that has gone unnoticed until now. The number of hornbills hunting was greater than that of bat hawks (*Machaeramphus alcinus*), a diurnal raptor specialized in hunting bats. Further systematic monitoring of these and other diurnal avian predators is necessary to fully understand the pressure they exert on bats.

## Graphical abstract

We observed a group of bushy‐crested hornbills ( *Anorrhinus galeritus* ) surprisingly hunting bats as they fly out of a large roosting cave in Bornean Malaysia, but not when they are roosting inside the easily accessible cave. The frequency and skill with which they captured bats in flight suggest that this is not just anecdotal evidence, but rather an underreported, commonly used hunting strategy.

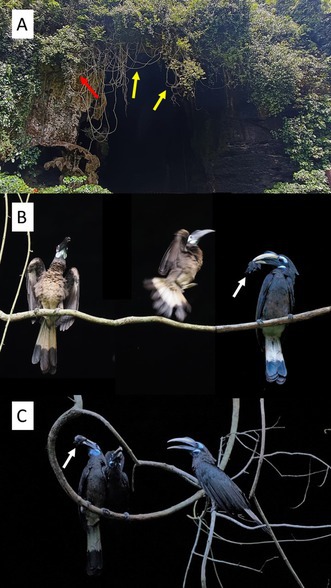

## 1 Introduction

While predation of bats by a wide variety of nocturnal and diurnal birds of prey has been reported worldwide, there are very few records of bats being preyed upon by non‐specialist predators such as hornbills (Mikula et al. 2016, 2024). The 54 species of extant hornbills are medium‐sized to very large birds (ranging in body mass from ca. 100 to 6180 g) that are widely distributed across Afrotropical, Indo‐Malayan, and Australasian regions, inhabiting a variety of habitats from the periphery of deserts to tall rainforest (Kemp 2001). While the two ground‐hornbill species are principally carnivorous, the rest of the group are omnivorous, eating a combination of animal and plant foods that vary among species and habitats. Most of the predominantly frugivorous species, such as the Asian species, live in forests, while species with more carnivorous tendencies tend to live in savannas (Kemp 2001). There is also a tendency for frugivorous species to become more carnivorous when breeding, preying on a variety of invertebrates and small‐sized vertebrates (Kemp 2001). By determining the prey brought to the nests, it was found that two of the four species of hornbills studied in Thailand consumed, albeit anecdotally, small insectivorous bats (Poonswad et al. 1998). Thanks to additional sporadic observations, 12 hornbill species have been reported to feed on bats (Mikula et al. 2016; Amal et al. 2021). However, the methods hornbills use to capture bats are not well known. Bats can be captured while they are roosting during the day in caves or trees, when they are more vulnerable to non‐specialized predators such as hornbills, or they can be hunted in flight when emerging from their roosts, as some agile aerial‐hunting birds of prey do (Mikula et al. 2016, 2024).

In this paper, we detail multiple observations of a group of bushy‐crested hornbills ( *Anorrhinus galeritus* ) hunting bats as they fly out of a large roosting cave in Bornean Malaysia, but not when they are roosting inside the easily accessible cave. The frequency and skill with which this medium‐sized hornbill species (length 65–70 cm, body mass 1134–1247 g; Kemp 2001) captured bats in flight suggest that this is not just anecdotal evidence, but rather an underreported, commonly used hunting strategy.

## 2 Study Site

The Gomantong Caves are an intricate cave system protected by the Gomantong Forest Reserve, a 51 km^2^ lowland forest that is the largest intact component of the 260 km^2^ Kinabatangan Wildlife sanctuary, sited in Sandakan Division, Sabah, Malaysia. Simud Hitam (5.530550 N, 118.071734 E) is the more accessible of the two caves and it is open to the general public though its main entrance. Its ceiling can reach 40–60 m high, where there is the “Great Light Hole.” Although up to 12 species of bats have been reported in Gomantong Forest Reserve, the bat population is dominated by a colony of the wrinkle‐lipped free‐tailed bat (*Mops plicatus*, previously *Chaerephon plicatus* ), a medium‐sized species (body mass 10.5–18 g; Phillipps and Phillips 2020a) that roost inside the Simud Hitam cave, reaching about 276,000 individuals in 2012 (McFarlane et al. 2015). This colony begins exiting the roost approximately an hour before sunset, with most bats flying in an anti‐clockwise spiral pattern within the “Great Light hole,” to finally leave this big hole in a consistent stream (McFarlane et al. 2015). A 30‐min climb along a path with stairs leads to an observatory adjacent to the light hole, which attracts tourists to observe the continuous stream of wrinkle‐lipped free‐tailed bats emerging from the hole. However, although less conspicuously and less known to the public, we observed that several thousand bats also emerge from the cave through the main entrance. We took photographs with an OM System OM‐1 Mark II camera and M. Zuiko Digital 150–400 mm TC1.25 zoom lens, and a short video with a Xiaomi 13T Pro phone.

## 3 Observations

**FIGURE 1:**
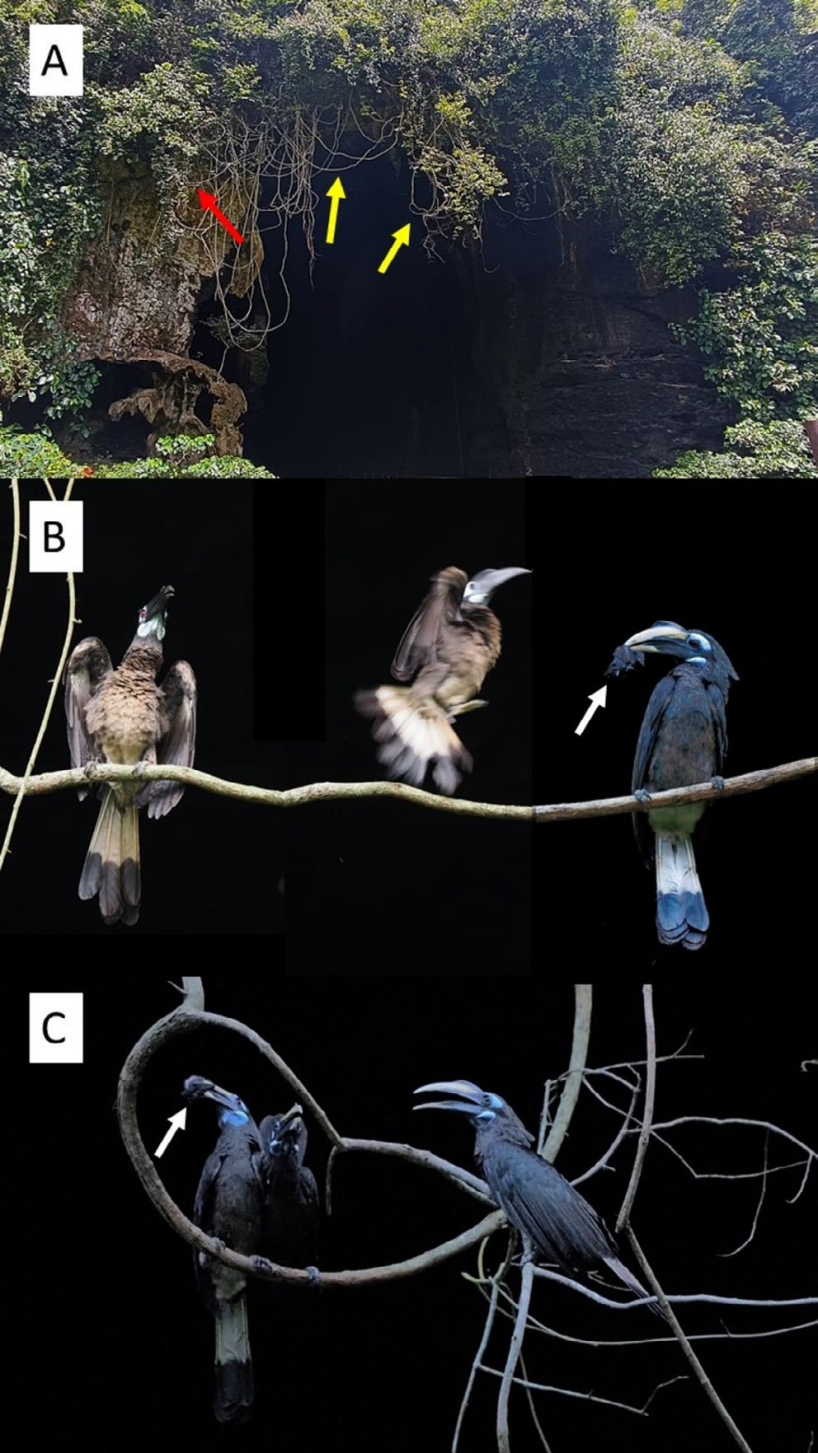
Main entrance of the Simud Hitam cave (A), indicating the perches where bushy‐crested hornbills were photographed (yellow arrows) and videotaped (red arrow) hunting bats in flight by jumping and short flights (B) or perching, waiting for bats that fly by at short distances, catching them with quick movements of the beak (C). The white arrows indicate recently captured bats, before they were quickly swallowed. Photographs: C.B. Sánchez‐Prieto (A) and J.L. Tella (B and C).

On July 14, 2024, we visited the interior of Simud Hitam cave and remained in the vicinity of its main entrance, recording all birds and mammals present between 10:43 and 14:00 h. As the only predator observed, an adult crested serpent eagle ( *Spilornis cheela* ) remained perched on the dry branches hanging over the cave entrance (Figure 1A) for over an hour, then entered the cave, but we did not see it leave. We also saw a female wreathed hornbill ( *Rhyticeros undulatus* ) flying around.

We returned to the cave at 15:45 and remained recording all observed species until 18:40, splitting into two groups to simultaneously monitor the main cave entrance and the light hole. At approximately 17:30, two bushy‐crested hornbills flew in and perched on the dry branches and vines hanging from the main entrance (Figure 1A), close to the crested serpent eagle, which again remained perched there most of the time. Shortly thereafter, more bushy‐crested hornbills arrived and began attempting to hunt the first bats emerging from the main entrance. These hornbills successfully used two hunting tactics. The first consisted of jumping from perches and making short flights of 2–3 m, attempting to capture bats flying past them with their beaks (Figure 1B). The second tactic consisted of remaining motionless and, through agile beak‐stretching movements, sometimes turning 180° with a short hop from the same perch, catching bats that flew by within a meter of the hornbills (Figure 1C, Video 1). It seems that the second tactic was used more as time went on and the flow of bats coming out was much greater (Video 1). In both cases, the bats were squeezed with the tips of their beaks and swallowed whole in a matter of seconds. The speed with which everything happened, while we tried to capture the events by taking photographs in already poor lighting conditions, made it difficult to assess the hunting success, the total number of bats caught, and the number of hornbills involved. During the short video we took with a smartphone, which lasts only 65 s, two hornbills were recorded using the second hunting tactic. One of them tried two times without success, while the other managed to catch a bat after eight failed attempts (Video 1). We observed up to eight hornbills successfully hunting bats at the same time (four adult females, three adult males, and one immature), but we cannot rule out the possibility that there were more or that there could be a turnover of individuals, with some being satiated and leaving while others arrived.

**VIDEO 1:** Two bushy‐crested hornbills ( *Anorrhinus galeritus* ) hunting wrinkle‐lipped free‐tailed bats (*Mops* *plicatus*) as they fly out of the main entrance of the Simud Hitam cave, July 14, 2024. Video: J.L. Tella. Video content can be viewed at https://onlinelibrary.wiley.com/doi/10.1002/ece3.71744.

Throughout our afternoon observation period, we also observed a male wrinkled hornbill (*Rhabdotorrhinus corrugatus*) flying past the main entrance of the cave. As with the crested serpent eagle, it did not show any intention of hunting flying bats. We did not observe any other potential predator species at the main entrance. At the same time, we observed the largest exodus of bats leaving the cave through the light hole, where we did not observe any hornbills but did record other species of specialized avian predators. At least five bat hawks (*Machaeramphus alcinus*), an adult pair of peregrine falcons ( *Falco peregrinus ernesti* ), and an adult Wallace's hawk‐eagle ( *Nisaetus nanus* ) attempted to hunt in the huge column of bats that emerged through the light hole. Peregrine falcons successfully hunted on several occasions, perching on a dead tree to consume the bats. The bat hawks, however, consumed the bats while flying.

## 4 Discussion

Here we report the first case of bat predation by bushy‐crested hornbills, contributing to the scarce information available in the literature on hornbills feeding on bats (Poonswad et al. 1998; Mikula et al. 2016; Amal et al. 2021). But more importantly, we were able to observe the different strategies used by hornbills to hunt bats. The hornbills did not enter the cave in the morning or afternoon, when thousands of bats roost, perched on the cave ceiling, and may be more easily accessible to non‐specialized predators. This strategy may have been that of the crested serpent eagle, which entered the cave in the morning and has been previously reported to hunt wrinkle‐lipped free‐tailed bats (Mikula et al. 2016). However, all of the hornbills' hunting activity was focused on bats that flew out of the cave at dusk. We find it surprising that these hunting scenes have not been reported before. Gomantong Caves, and especially Mulu Caves, also in Bornean Malaysia, are popular tourist attractions due to the spectacular exodus of thousands of bats at dusk, but are also known among biologists and birdwatchers because of the presence of bat hawks and Wallace's hawk‐eagles hunting these bats (Phillipps and Phillips 2020a). Moreover, hornbills attract attention by themselves by their large size, massive bills, conspicuous behavior, and loud calls (Kemp 2001).

The hunting behavior described here may have been observed but not reported in the scientific literature. Nonetheless, this behavior may have gone largely unnoticed, since, as we were able to corroborate in both Gomantong and Mulu caves, tourists and birdwatchers gather at the observatories from where the mass exodus of bats can be seen emerging in swarms that gradually fade into the sky. These large groups of bats also attract agile aerial hunters, such as bat hawks, which are easily observed by people flying in the sky. However, hornbills hunt bats in flight but in ambush from perches far from the view of tourists and birdwatchers.

It has traditionally been assumed that among avian predators, only owls and the bat hawk, a diurnal Old World bird of prey specialized in hunting bats at dusk, exert significant predation pressure on bats (Kemp 2001). Recent studies show several other species of diurnal raptors hunting bats efficiently (Brighton et al. 2021). Much less well known is the role of a wide variety of opportunistic species that apparently hunt bats only anecdotally (Mikula et al. 2016, 2024). Our observations, although apparently anecdotal, show how three specialized diurnal raptor species were altogether as abundant as a single species of hornbill hunting bats in flight. More attention should be paid to the role of the wide variety of opportunistic predators, since together with specialized predators they can make the risk of predation several orders of magnitude higher during the day than at night (Mikula et al. 2024). Indeed, pressure exerted by diurnal avian predators may affect the emergence patterns of bats from their roost sites (Arndt et al. 2018; Mikula et al. 2024), and could even explain their strictly nocturnal behavior (the “avian predator hypothesis,” Rydell and Speakman 1995; Mikula et al. 2024).

Our observations raise several questions. What is the actual hunting pressure exerted by both bushy‐crested hornbills and diurnal raptors in terms of the number of bats hunted per day? Do these predation pressures vary seasonally? Are these bushy‐crested hornbills a small group of individuals specialized in hunting bats, or is this a generalized behavior involving numerous individuals taking turns? Do only bushy‐crested hornbills hunt bats, or are there other hornbill species that also do so but were not observed by us? During 2 weeks of field observations, we recorded 105 hornbills of the 8 species present in the region (Phillipps and Phillips 2020b), of which bushy‐crested hornbills were the most frequently observed (48% of individuals). Therefore, other species of hornbills could also be hunting bats but may be more difficult to observe given their lower abundances. However, we observed two other species of hornbills in the vicinity of the main entrance, which did not approach or attempt to hunt bats. It is possible that these other species do not hunt bats or do so in a more anecdotal way. In fact, it is known that each hornbill species has specific food and foraging preferences, and vertical niche partitioning allows for multiple species to coexist within the same habitats (Kemp 2001). All of these questions could be easily answered, given the easy accessibility of the Gomantong caves, by longer‐term monitoring of the number and species of avian predators and their success in hunting bats across seasons.

Finally, the question arises as to why hornbills hunt bats in flight when it appears to be energetically more demanding than feeding on fruit. It has been previously suggested that some hornbill species tend to feed more on animals during breeding, as a response to an increased protein requirement (Kemp 2001). It is also possible that the availability of fruits varies seasonally and that hornbills exploit bat emergence from roosts as a predictable and energetically profitable food source throughout the year, as do other avian predators (Mikula et al. 2024). These hypotheses could be tested by monitoring fruit availability and hornbill hunting behavior throughout the breeding and non‐breeding seasons.

## Author Contributions

**José L. Tella:** conceptualization (equal), data curation (equal), investigation (equal), visualization (lead), writing – original draft (lead), writing – review and editing (equal). **Cristina Fuentes‐Sendín:** conceptualization (equal), investigation (equal), writing – review and editing (equal). **Carlos Gutiérrez‐Expósito:** conceptualization (equal), investigation (equal), writing – review and editing (equal). **Gema Ruiz‐Jiménez:** conceptualization (equal), investigation (equal), writing – review and editing (equal). **Raquel Sainz‐Elipe:** conceptualization (equal), investigation (equal), writing – review and editing (equal). **Cristina B. Sánchez‐Prieto:** conceptualization (equal), investigation (equal), writing – review and editing (equal). **David Serrano:** conceptualization (equal), investigation (equal), writing – review and editing (equal).

## Conflicts of Interest

The authors declare no conflicts of interest.

## Data Availability

All the information obtained from our observations is provided in the manuscript.
